# Nitric oxide synthase 2 (NOS2) expression in histologically normal margins of oral squamous cell carcinoma

**DOI:** 10.4317/medoral.19351

**Published:** 2013-12-07

**Authors:** Rosana Morelatto, María E. Itoiz, Natalia Guiñazú, Daniel Piccini, Susana Gea, Silvia López-de Blanc

**Affiliations:** 1 DDS, PhD Assistant Professor, Clinical Stomatology “B”. School of Dentistry National University of Cordoba; 2 DDS, PhD Emeritus Professor, School of Dentistry, University of Buenos Aires; 3 DCS, PhD Assistant Professor. Department of Chemical Biochemistry. School of Chemical Sciences. National University of Cordoba; 4 DMS, PhD Head Professor, Pathology Service. School of Medicine. National University of Cordoba; 5 DCS, PhD Associated Professor, Department of Chemical Biochemistry. School of Chemical Sciences. National University of Cordoba; 6 DDS, PhD Head Professor. Clinical Stomatology “B”. School of Dentistry. National University of Cordoba, Cordoba. Argentina

## Abstract

The activity of Nitric Oxide Synthase 2 (NOS2) was found in oral squamous cell carcinomas (OSCC) but not in normal mucosa. Molecular changes associated to early carcinogenesis have been found in mucosa near carcinomas, which is considered a model to study field cancerization. The aim of the present study is to analyze NOS2 expression at the histologically normal margins of OSCC. Study Design: Eleven biopsy specimens of OSCC containing histologically normal margins (HNM) were analyzed. Ten biopsies of normal oral mucosa were used as controls. The activity of NOS2 was determined by immunohistochemistry. Salivary nitrate and nitrite as well as tobacco and alcohol consumption were also analyzed. The Chi-squared test was applied. Results: Six out of the eleven HNM from carcinoma samples showed positive NOS2 activity whereas all the control group samples yielded negative (*p*=0.005). No statistically significant association between enzyme expression and tobacco and/or alcohol consumption and salivary nitrate and nitrite was found. Conclusions: NOS2 expression would be an additional evidence of alterations that may occur in a state of field cancerization before the appearance of potentially malignant morphological changes.

** Key words:**Field cancerization, oral squamous cell carcinoma, Nitric Oxide Synthase 2 (NOS2), malignity markers.

## Introduction

Despite the fact that the oral cavity is an accessible location for medical examination, most cases of oral cancer (OC) are detected at advanced stages, which is the reason for the low survival rates recorded. OC has high morbidity rates, with an overall survival rate of 34-56% (1,2). In Cordoba, Argentina, mortality rates have increased noticeably in females, 77% for the period 1975-2000 (3). These tendencies probably indicate a change in women´s habits, such as increase in smoking and alcohol drinking (4-6). In a previous study, we found that late diagnosis is mainly due to professional delay in indicating a biopsy (7). Loco-regional recurrence is the main reason for the failure of head and neck cancer treatments. Failure is associated to the remaining cancer cells in the surgical margins that are considered negative in the pathologically examined sample (8). This could be explained a lack of sensitivity of the method used to identify cells that have already started their malignity transformation and have not yet developed a pathological phenotype. Besides Slaughter, introduced the concept of “field cancerization” to explain the increased risk of malignant transformation in large areas of the epithelial lining of the upper aerodigestive tract, modified by tobacco and alcohol consumption (9). This hypothesis was based on the high incidence of second primary tumors or multifocal cancer and was proved by the demonstration of molecular changes in clinically healthy mucosa of smoking patients (10,11). Furthermore, the sequential or simultaneous development of oral premalignant and/or malignant lesions in a single patient evidences progressive genotypic and phenotypic alterations associated to field cancerization (12). The search for markers of field cancerization before the appearance of premalignant morphological alterations is of biological interest and clinically relevant in terms of early diagnosis and OC prevention. We have tried to detect a field cancerization by means of immunohistochemical (IHC) reactions, easy to apply to routine biopsic material (13-16).

Nitric oxide (NO) is a small, relatively stable, free radical gas, found both in normal and in malignant tissues (17,18). It is synthesized by nitric oxide synthases (NOS) which exists in three different isoforms: neuronal NOS (NOS1), endothelial NOS (NOS3), and inducible NOS (NOS2). Lipopolysaccharide, interferon and numerous other factors induce NOS2 expression in endothelial and inflammatory cells (19,20). NOS2 is also expressed in some normal epithelia such as airway epithelium, basal keratinocyte layer of normal skin, and normal salivary ducts (21). Neither NOS2 protein nor mRNA was found in normal oral mucosa (22). Neoplastic tissues, including head and neck carcinomas, over-express the enzyme. NOS2 has been involved in tumor growth, mutagenicity, angiogenesis and metastasis (23,24). NOS2 activity was also found in oral epithelial dysplasia, submucous fibrosis and verrucous hyperplasia (25).

Considering that alterations associated to field cancerization have been found in normal epithelia near oral carcinomas (11-14,16), the present study focuses on the evaluation of the NOS2 expression in these areas as another biomarker for risk of malignant transformation. Since NOS2 enzyme intervenes in NO synthesis and given the fact that NO is highly reactive, saliva levels were determined by measuring nitrate and nitrite, which are the NO oxidation products (26,27).

## Material and Methods

Tissue sources:

Out of eleven biopsy and/or excised surgical archive specimens of oral squamous cell carcinomas (OSCC), only samples involving histologically normal oral mucosa were selected, provided they were situated at more than 5mm away from the tumor without or with very mild inflammatory infiltrate.

Ten specimens of clinically and histologically normal oral mucosa obtained during surgery for deep seated benignant lesions were selected as control group (CG). All the samples belonged to patients attending the Oral Pathology Department, Facultad Odontología, Universidad Nacional de Córdoba. All the cases included presented complete clinical records and saliva complementary studies, as well.

Exclusion criteria were pregnancy, use of vitamin supplements, antibiotics or anti-inflammatory agents, and previous oncological treatment.

Due to the well known influence of tobacco and alcohol in the development of a sub-clinic level of field cancerization, patients with a history of more than 200000 cigarettes or with more than 50g of alcohol per day were discarded (28).

A complete clinical record included the following data.

Clinical stage of patients: According to the Union for International Cancer Control (UICC) criteria, stages III and IV were defined as advanced tumors and stages I and II as early ones (29).

Tobacco consumption: The total number of cigarettes smoked during the patient’s lifetime was estimated according to Zanetti *et al*. method (30), multiplying the number of cigarettes smoked per year by the number of years. Patients were categorized as: non-smokers (never having smoked), or smokers (having smoked more than 100,000 cigarettes).

Alcohol consumption: one alcoholic unit per day (1U = 8 to 10 g of ethanol = 1 glass of wine = 1/4 liter of beer = 1 measure of spirits) was considered alcohol exposure according to Pentenero *et al*. 2008 (31). The total alcohol intake was estimated considering the grams per day thus obtaining the approximate lifetime alcohol consumption. Patients were categorized according to their consumption as: non-drinkers (never having drunk) and drinkers (having consumed more than 100,000 g).

Concentration of nitrate and nitrite in saliva samples: Samples were obtained and processed following laboratory standardized conditions.

Nitrite levels were considered low or high according to whether concentrations were lower or higher than 100 µM respectively, while nitrate levels were considered low or high according to whether concentrations were lower or higher than 800 µM, respectively.

Immunohistochemistry: Sections of 7µm thick were obtained; endogenous peroxidase was blocked by immersion in 0.5% methanolic peroxide for 15 min. Antigen retrieval was performed with Citra (Biogenex) in 3 cycles of 4 minutes each, at 400W microwaves. The sections were then incubated overnight with the primary antibody (anti-NOS2, C-terminal, rabbit, Cat. sc-651, Santa Cruz Biotechnology, Inc.), diluted 1:50 in PBS in a humid chamber at 4ºC. Antibody binding sites were visualized using a streptavidin-peroxidase detection kit (Kit Multilink, Biogenex). Slides with and without hematoxylin counterstain were mounted. As positive control a section of a single block of apical granuloma was included in each staining batch, rendering highly positive for NOS2. As negative control for antibody specificity tissue samples were included in which the primary antibody was omitted.

The presence of brown precipitate in the cytoplasm was considered positive provided it could be clearly distinguished from the negative reaction of the nucleus in preparations without counterstaining.

In each case the staining intensity was evaluated by comparing it with the respective positive control of the same staining batch. A reaction evident but less intense than the control, was considered mildly positive; while a reaction similar or higher than the control, was considered positive or highly positive respectively. In the present study the tumor epithelial cords and their histologically normal margins were grouped in positive (+) or negative (-), independently from the intensity of the reaction. Two oral pathologists, who ignored the clinical data, evaluated the immuno-histochemical assays.

Statistical analysis: A Chi Square test was applied to evaluate the differences in NOS2 expression, between the histologically normal margins of OSCC and the control group, and between tobacco and alcohol consumption and salivary nitrate and nitrite concentrations.

## Results

Sample features.

In this cross-sectional study 21 samples were examined: 11 OSCC samples corresponding to the study group and 10 samples as control group. No statistically significant association was found regarding tobacco and alcohol consumption in both groups.  Table 1 shows the characteristics of the included cases.

**Table 1:**
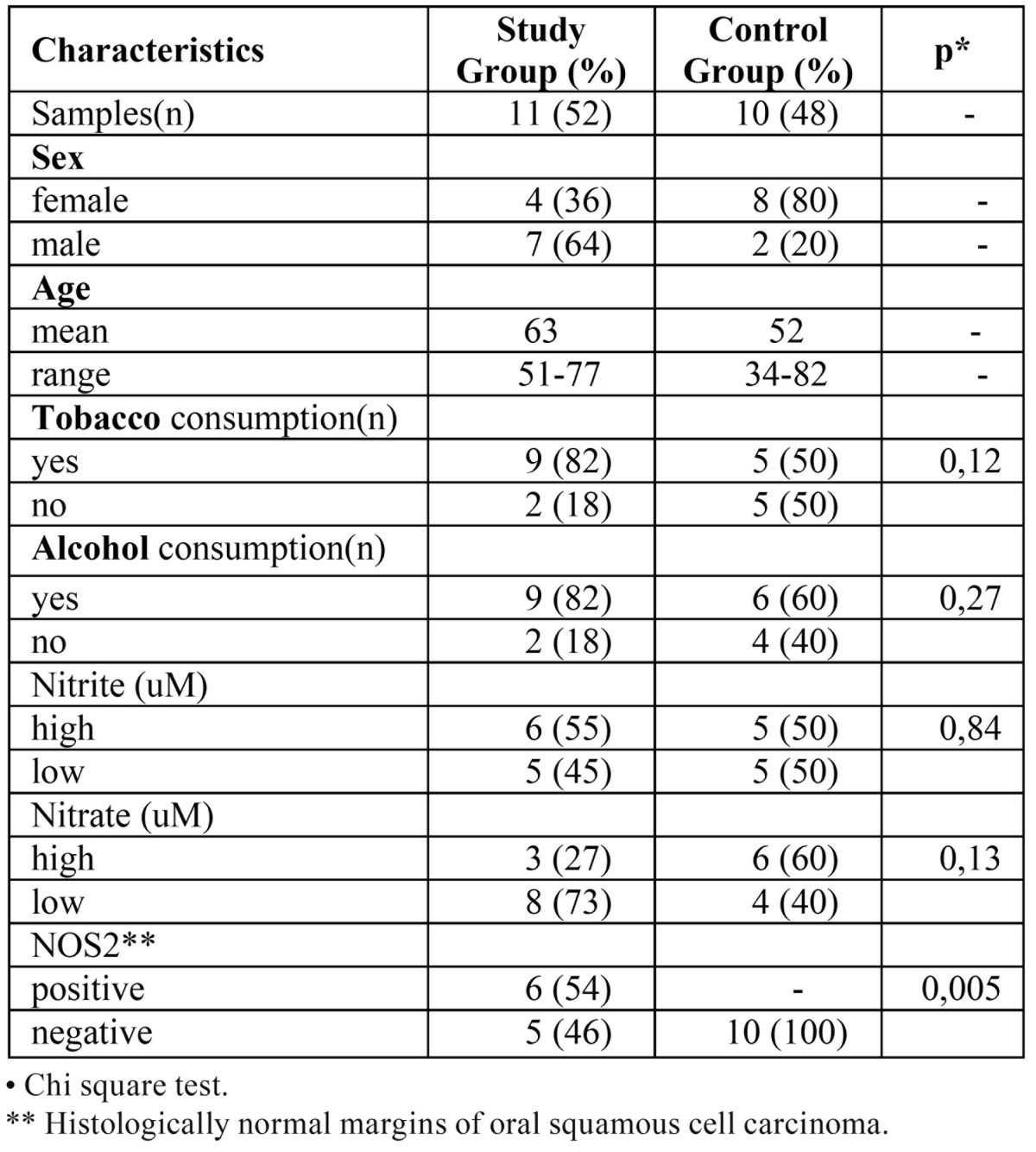
Characteristics of the study and control group.

NOS2 citoplasmatic expression in OSCC

**Figure 1:**
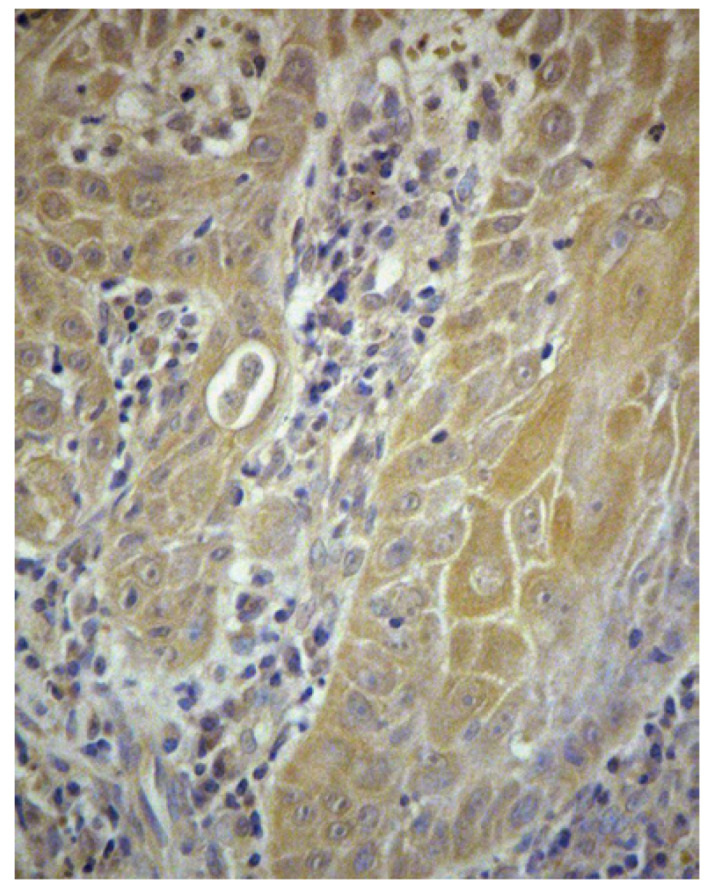
Immunohistochemical expression of NOS2 in epithelial cords of OSCC. Haematoxilin counterstain (x25).

NOS2 activity is expressed in the cellular cytoplasm as a diffuse precipitate without any nucleous reaction. Carcinoma reactivity does not differ from the existing one in the literature (32). A different intensity reaction was observed in the OSCC epithelial cords, in the stroma inflammatory infiltrate and in the vascular endothelium (Fig. 1).

**Figure 2:**
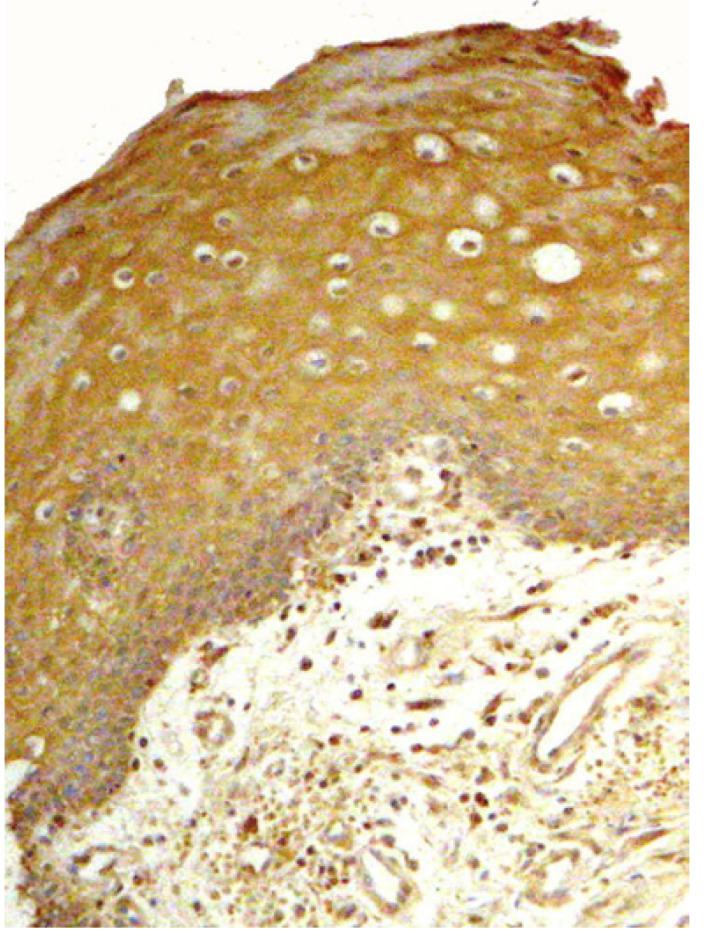
NOS2 expression in histologically normal margins of OSCC. Endothelial and inflammatory cells were also positive (x10).

Histologically normal mucosa of the tumor margins showed reactions ranging from negative to positive expressions in all epithelial layers (Fig. 2). Six out of the 11 cases under study were positive while in the CG the epithelium was negative for NOS2 reaction in all cases (*p*= 0.005). The Odds Ratio (OR) could not be calculated since the control group did not express NOS2 in any sample; therefore, the corresponding frequency was zero (0).

Only a moderate NOS2 expression in the connective tissue was found in both groups whenever isolated inflammatory cells were present.

 Table 2and  Table 3 show the characteristics of the NOS2 expression in study and those of the control group, respectively.

**Table 2:**
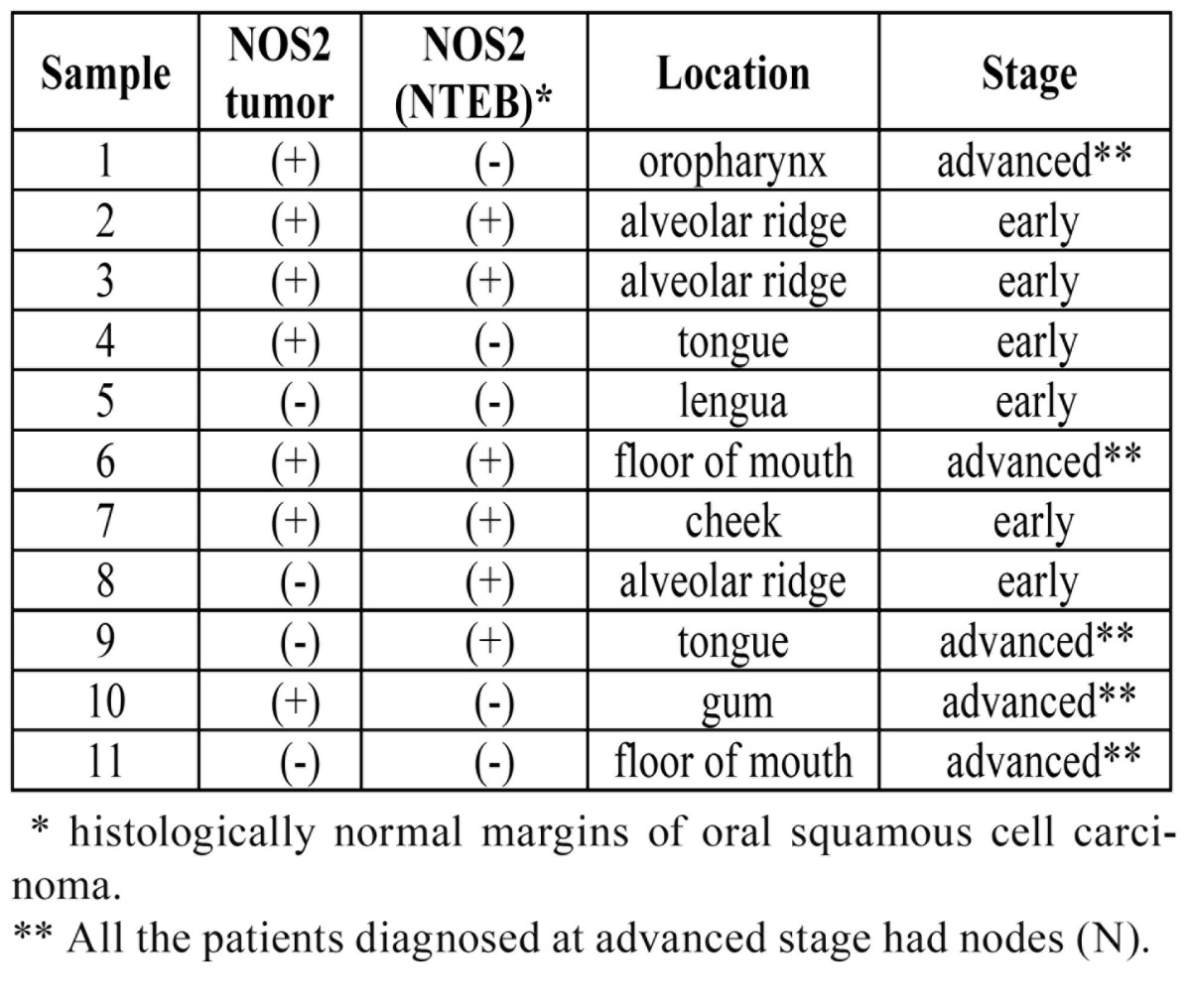
Study Group (OSCC): NOS2 expression, tumor stage and location.

**Table 3:**
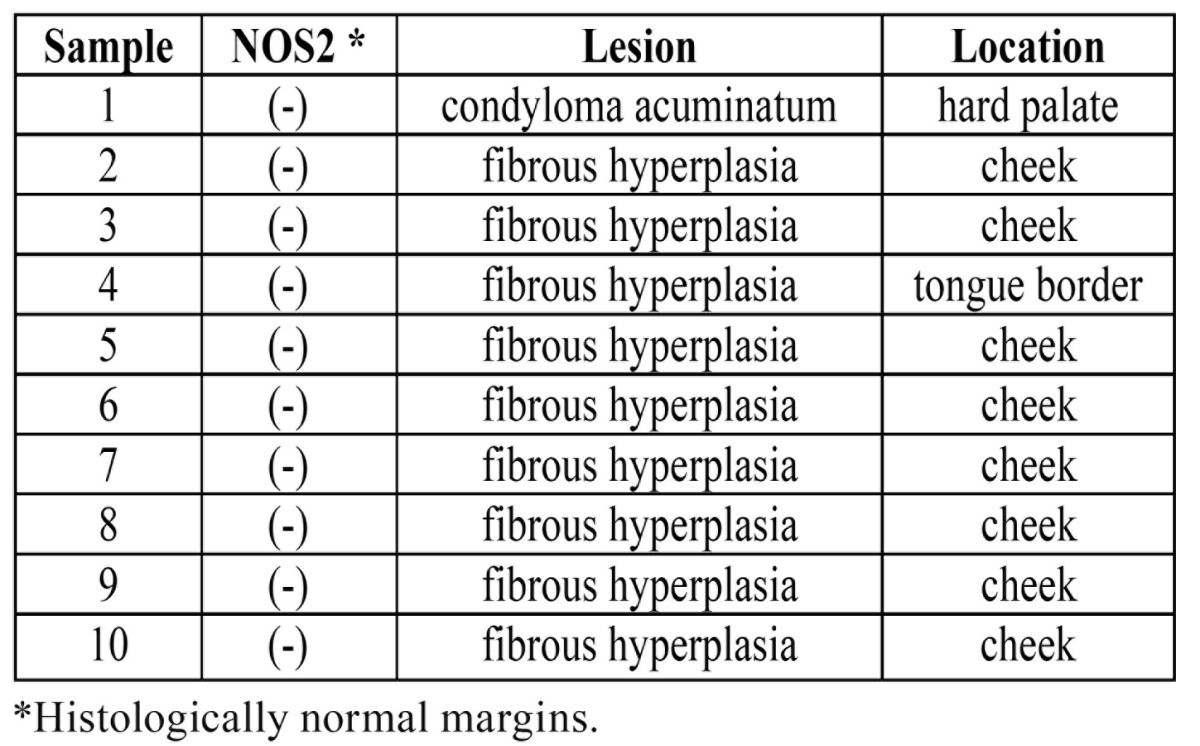
Control Group: NOS2 expression, lesion and location.

Nitrite and nitrate levels

The salivary nitrate and nitrite levels were similar both in carcinoma and control groups (see  Table 1).

## Discussion

Tumor epithelial cords were positive for NOS2; the degrees of expression were variable, in agreement with previous studies (32). Activation of NOS2 has also been demonstrated in lung, colon and prostate cancer and in melanoma (25,33-36) and has been related to cervical lymph node metastasis and to greater metastatic potential (25,32). Over-expression of NOS2 has been found in dysplasia specimens (17,33,37) and has also been involved in early cellular changes leading to malignity such as transformation of normal cells or growth of altered cells (36).

The present study not only analyzed NOS2 expression in tumor tissues but also demonstrated its activity in the surrounding normal tumor epithelium. This is the first time in which a NOS2 remarkable expression is reported for the histologically normal mucosa surrounding carcinomas. In these still morphologically unaltered areas, genetic and histochemical alterations typical of a field cancerization have been proven (9,10).

Since NOS2 expression is triggered by inflammatory reactions, and with the aim of determining if its expression in OSCC normal margins is independent from the inflammatory response, we only included samples bearing the inevitable presence of some isolated inflammatory cells in both study and control groups. The strict inclusion criteria limited the number of samples available.

It is well known that the sum of genetic alterations lead to gradual phenotypic changes. In this work, some positively stained tumors turned out to have negative margins. Conversely, some negative tumors were found to have positive margins. The complexity and the heterogeneity of the NOS2 expression in tumors and within a same tumor has been found in the literature (36); the same finding was reported when other cancerization markers have been used (12,15). It has been suggested that NOS2, which is over expressed in transformed cells, can mediate endothelial proliferation through NO which, in turn, acts as a cellular signal for angiogenesis (25). We found an increased NOS2 expression in the OSCC endotelial cells. Interestingly, we had previously demonstrated a rise in sub-epithelial capillaries of dysplastic leukoplakia, non-invasive margins of carcinoma (15) and oral mucosa of alcoholic patients (16). Moreover, NO has been suggested to modulate different cancer-related events including angiogenesis, apoptosis, cell cycle, invasion and metastasis (20). The anti-tumoral NO effects described in other research works stimulates apoptosis and inhibit angiogenesis (17,18,38), although further clinical studies of cancer patients are still necessary. The biological activity of NO depends on variables such as the originating source, its local concentration, tumor microenvironment and spatial and temporal constants that may determine the ON function.

More studies are necessary to establish the role of the path ON/NOS2 in tumor genesis and evolution and to determine the usefulness of NOS2 inhibitors as chemo-prevention medication (39,40).

The levels of salivary nitrite and nitrate were measured looking for their relationship with the expression of NOS2 in patients with tumors. No differences were found with the control group. Taking into account the fact that nitrite concentration in saliva is associated with age, diet composition, fungal or bacterial infection and with tobacco and alcohol consumption, we find it important to continue enlarging our sample in the search for a possible association.

NOS2 expression in clinically and histologically normal mucosa would be an additional evidence of the alterations that may occur in a field of cancerization before potentially malignant morphological changes appear.

This preliminary data suggest that the expression of NOS2 could be useful for the diagnosis of “field cancerization” or “at risk mucosa” in those patients exposed to risk factors like excessive tobacco and alcohol consumption but who have not yet evinced lesions in the oral mucosa.
